# Advances in IKBKE as a potential target for cancer therapy

**DOI:** 10.1002/cam4.2678

**Published:** 2019-11-15

**Authors:** Min Yin, Xin Wang, Jie Lu

**Affiliations:** ^1^ Department of Oncology Jinan Fifth People's Hospital Jinan PR China; ^2^ Department of Oncology Renmin Hospital of Wuhan University Hubei Province Wuhan PR China; ^3^ Department of Radiation Oncology Shandong Cancer Hospital Affiliated to Shandong University Shandong Academy of Medical Science Jinan PR China; ^4^ Department of Neurosurgery The First Affiliated Hospital of Shandong First Medical University Jinan PR China

**Keywords:** IKBKE, NF‐κB, signaling pathway, small molecule inhibitors

## Abstract

IKBKE (inhibitor of nuclear factor kappa‐B kinase subunit epsilon), a member of the nonclassical IKK family, plays an important role in the regulation of inflammatory reactions, activation and proliferation of immune cells, and metabolic diseases. Recent studies have demonstrated that IKBKE plays a crucial regulatory role in malignant tumor development. In recent years, IKBKE, an important oncoprotein in several kinds of tumors, has been widely found to regulate a variety of cytokines and signaling pathways. IKBKE promotes the growth, proliferation, invasion, and drug resistance of various cancers. This paper makes a detailed review that focuses on the recent discoveries of IKBKE in the malignant tumors, and puts forward that IKBKE is becoming an important therapeutic target for clinical treatment, which has been more and more realized.

## Graphical abstract

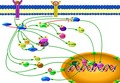

## 1 INTRODUCTION

The IKK (IκB kinase) family, whose members are key activators of the NF‐κB signaling pathway, plays an important role in the regulation of NF‐κB‐mediated inflammatory reactions, immune cell activation, and tumorigenesis. The IKK family mainly consists of five protein factors: IKKα, IKKβ, IKKγ (also known as NEMO), IKBKE, and TBK1. Recent studies demonstrated that IKBKE, as an IKK family protein factor, plays an important regulatory role not only in the activation of inflammatory factors and the progression of metabolic diseases and cellular immunity but also in the development of various malignant tumors.

IKBKE (IKKε, also known as IKK‐i) is a serine/threonine protein kinase belonging to the IKK family and has a molecular weight of 80 kDa. The IKBKE gene is located at 1q32 and has 22 exons. In 1999, Shimada et al^1^ first isolated a novel kinase called IKK‐i that was induced by LPS (lipopolysaccharide) in mouse macrophage cell lines using the SSH (suppression subtraction hybridization) technique. Furthermore, IKK‐i was found to be highly expressed in normal pancreatic tissue, thyroid tissue, spleen tissue, and peripheral blood leukocytes. IKBKE expression was upregulated by stimulation with LPS or other inflammatory cytokines, such as TNF‐α, IL‐1, IL‐6, and IFN‐γ. Subsequently, Peters et al^2^ discovered the new protein IKBKE induced by PMA (phorbol 12‐myristate 13‐acetate) and confirmed it as IKK‐i by analyzing its amino acid sequence, which is distinct from those of IKKα, IKKβ, and IKKγ. Nevertheless, determination of the amino acid sequence revealed 33% and 31% homology with IKKα and IKKβ, respectively, and 67% homology with TBK1 (TANK‐binding kinase 1),^3^ which has a similar HLH (helix‐loop‐helix) structure at the C‐terminal region of the protein and has an LZ (leucine zipper) domain similar to the middle region of IKBKE. Ikeda et al^4^ demonstrated that the adjacent kinase activity domain of IKBKE has a ULD (ubiquitin‐like domain) motif similar to IKKβ to maintain downstream kinase activity and to regulate downstream signaling. However, IKBKE lacks the NBD (NEMO‐binding domain) at the C terminus of the protein, unlike IKKα and IKKβ, and thus cannot form the IKK complex with IKKγ (NEMO) to induce activation of the NF‐κB signaling pathway.

The classical NF‐κB signaling pathway is triggered by a kinase complex that includes IKKα and IKKβ as catalytic subunits and the scaffold protein NEMO, which phosphorylates protein factors of the NF‐κB signaling pathway. Similar to IKKα and IKKβ, IKBKE and TBK1 are required to form kinase complexes with the help of scaffold proteins, including TANK (TNF receptor‐associated factor family member‐associated NF‐κB activator), NAP1 (NAK‐associated protein 1) and INTBAD (similar to NAP and TBK1 adaptor), to effectively stimulate their substrates.^5^, ^6^, ^7^ As a TRAF‐interacting protein, TANK (also known as I‐TRAF) synergizes with TRAF2^8^ and IKBKE/TBK1^9^ to trigger the NF‐κB signaling pathway. TANK could be regulated by the kinase complex, including TRAF3, and be phosphorylated by IKBKE/TBK1, thereby inducing IKBKE/TBK1‐mediated Lys^63^‐linked polyubiquitination and then activating downstream kinase complexes or pathways.^10^ Through analysis of the amino acid sequence, NAP1 was determined to share 28% homology with TANK. Fujita et al^11^ used an IP (immunoprecipitation) technique to demonstrate that NAP1 directly interacted with TBK1 and its family member IKBKE to form a protein kinase complex to effectively phosphorylate downstream factors of the NF‐κB signaling pathway, thus protecting cells from apoptosis by promoting NF‐κB activation. In contrast to TANK and NAP1, SINTBAD structurally binds to IKBKE/TBK1 but does not have kinase activity. Ryzhakov et al^12^ also demonstrated a conserved TBK1/IKBKE‐binding domain (TBD) in all three adaptor proteins, TANK, NAP1, and SINTBAD. In 2010, Koop et al^13^ identified two novel splice variants of human IKBKE and designated them IKKε‐sv1 and IKKε‐sv2. The gene encoding IKKε‐sv1 lacks exon 21, which leads to a deficiency of 25 amino acids at the C terminus. This mutation causes IKBKE to lose the ability to activate the downstream factor IRF‐3, but IKBKE keeps the capacity to stimulate the NF‐κB signaling pathway. The other mutation, IKKε‐sv2, which lacks exon 20, results in a 13 amino acid loss at its C terminus, thus leading to a deficiency in IRF‐3 stimulation and a loss of NF‐κB signaling pathway activation. These findings highlight that the C‐terminal region of IKBKE is required for the phosphorylation and activation of downstream molecules.^14^

Previous studies have demonstrated that IKBKE/TBK1 is downstream of several factors or receptors, such as TLR3 (Toll‐like receptor 3), RIG‐1 (retinoic acid inducible gene 1), MDA5 (melanoma differentiation associated gene 5), and IFN‐β (interferon‐β). After viral infection and stimulation with double‐stranded RNA (dsRNA), IKBKE/TBK1 was activated, and it then phosphorylated the C‐terminal region of IRF‐3 and IRF‐7, thus triggering the formation of IRF‐3 or IRF‐7 homo‐ or heterodimers that could transplant into the nucleus to express targeted genes.^15^, ^16^, ^17^ Fujii et al^18^ used mass spectrometric analysis to demonstrate that Ser‐386, Ser‐396, and Ser‐402 sites of IRF‐3 were directly phosphorylated by activated IKBKE when innate immune cells were attached by virus, thereby promoting the transcription of related antiviral factors such as interferon type I.^19^, ^20^ Nevertheless, viral infection or dsRNA‐induced expression of IFN‐α and ‐β genes were intact in IKBKE‐deficient MEFs (mouse embryonic fibroblast cells).^17^ Meanwhile, McWhirter et al^21^ showed that TBK1 played a key role in the activation and nuclear translocation of IRF‐3 in MEF, considering that TBK1‐deficient mice were deprived of the expression of IRF‐3‐dependent genes such as IFN‐α, IFN‐β, and IP‐10.^20^, ^21^, ^22^ This phenomenon was probably because the content of IKBKE in MEFs is far less than that of TBK1. In other words, the expression discrepancy between TBK1 and IKBKE in MEFs may be a major rationale for this phenomenon.^22^ Interestingly, they also found that low IRF‐3 expression in TBK1‐deficient MEFs can be restored by implantation with wild‐type IKBKE instead of IKBKE mutants lacking kinase activity.^22^ This fact demonstrated the importance of IKBKE kinase activity in IRF‐3 activation. Besides, Siednienko et al^23^ demonstrated that a TLR (Toll‐like receptor) named MyD88 can inhibit IKBKE‐ but not TBK1‐induced activation of IRF‐3. This study provided insight into the mechanism discrepancy between IKBKE‐ and TBK1‐mediated induction of IRF‐3.

In recent years, an increasing number of studies have shown that the expression and regulation of IKBKE is not merely limited to the fields of innate immunity, inflammatory response, and metabolic diseases but that it also extends to the fields of oncogenesis, progression, transformation, and chemotherapeutic resistance of cancers.

## 2 THE REGULATORY ROLE OF IKBKE IN MALIGNANCIES

Recent studies have shown that IKBKE is highly expressed in a variety of malignant tumors (Table 1), and the difference in expression is related to the prognosis of patients. In most cases, high expression of IKBKE is associated with poor prognosis, but in a few cases, IKBKE may indicate a slightly better prognosis. Generally, IKBKE plays an important role in tumorigenesis, antichemotherapeutic properties, tumor metastasis and tumor microenvironment.

**Table 1:** The expression of IKBKE in cancers

Tumors	Specimen type	Methods	Expression	References
Breast cancer	Cell lines (n = 49)	Whole genome structural analyses	16.5% (8/49)	[25]
Breast cancer	Primary specimens (n = 30)	Whole genome structural analyses	33.3% (10/30)	[25]
Glioma	Primary specimens (n = 71)	ICH	58.3% (58/71)	[30]
Glioma	Primary specimens (n = 51)	ICH, WB	88.2% (45/51)	[31]
Esophageal squamous cell carcinomas	Primary specimens (n = 58)	ICH	84.5% (49/58)	[34]
Gastric cancer	Primary specimens (n = 1107)	ICH	13.6% (150/1107)	[35]
Hepatocellular carcinoma	Primary specimens (n = 222)	ICH	90.1% (200/222)	[37]
Pancreatic ductal adenocarcinoma	Primary specimens (n = 78)	ICH	64% (50/78)	[38]
Pancreatic ductal adenocarcinoma	Primary specimens (n = 21)	WB	81.0% (17/21)	[39]
Pancreatic ductal adenocarcinoma	Cell lines (n = 14)	WB	85.7% (12/14)	[39]
Ovarian cancer	Primary specimens (n = 96)	WB, qPCR	65.6% (63/96)	[45]
Ovarian cancer	Cell lines (n = 14)	WB	78.6% (11/14)	[45]
Endometrial cancer	Primary specimens (n = 52)	qRCR, gene expression analysis	meaningful	[47]
Prostate adenocarcinoma	Primary specimens (n = 107)	ICH	71% (76/107)	[51]
Non‐small cell lung carcinoma	Primary specimens (n = 98)	ICH	55.1% (54/98)	[54]
Squamous cell lung carcinoma	Primary specimens (n = 288)	ICH	70.8% (204/288)	[55]

## 3 BREAST CANCER

Eddy et al^24^ showed that IKBKE was highly expressed in human breast cancer specimens and several breast cancer cell lines, which hinted that IKBKE might regulate the growth of breast cancer in some ways. Boehm et al^25^ found that IKBKE was highly expressed in 30% of breast cancer specimens and 16.3% of breast cancer cell lines through analysis of integrative genomic approaches, thus first identifying IKBKE as a new oncogene in breast cancer. They also found that the tumor cell death rate was increased when they silenced IKBKE expression in breast cancer cell lines that harbor IKBKE amplifications, which highlighted the regulatory role of IKBKE in tumor cell proliferation. In addition, increased IKBKE expression was observed at the transcriptional and translational levels in numerous breast cancer cell lines and breast cancer specimens, but expression was not absolutely correlated with DNA copy number changes. This suggested that multiple mechanisms regulated IKBKE expression. Furthermore, Qin et al^26^ showed that IKBKE knockdown in human breast cancer cells using siRNA resulted in an obvious reduction of tumor cell proliferation, migration, and invasion. In addition, the tumor cell cycle was arrested at the G_0_/G_1_ phase after silencing IKBKE by flow cytometry analysis, while IKBKE knockdown did not seem to significantly affect apoptosis in breast cancer cells. Barbie et al^27^ found that IKBKE was overexpressed in breast cancer cell lines, including TNBC (triple‐negative breast cancer) cell lines that were negative for ER (estrogen receptor), PR (progesterone receptor), and HER2 (human epidermal growth factor receptor 2), and that knocking down IKBKE in TNBC cell lines decreased tumor cell proliferation and colony formation. Furthermore, treatment of IKBKE‐driven breast cancer cells with a potent inhibitor of TBK1/IKBKE and JAK signaling impaired proliferation and colony formation of TNBC cells, whereas inhibition of JAK alone did not, suggesting that IKBKE regulated tumor cell progression. Li et al^28^ demonstrated that IKBKE interacted with ER‐α36 (estrogen receptor‐α), a novel variant of ER‐α, and increased its expression in breast cancer cells, enhancing ER‐α36‐mediated mitogenic and nongenomic estrogen signaling. In addition, knocking down IKBKE also inhibited the proliferation of TNBC cells. Tang et al^29^ showed that the upregulation of IKBKE suppressed taxol‐induced apoptosis and led to increased resistance to taxol (paclitaxel). Moreover, the expression of IKBKE was positively correlated with T stage, lymph node metastasis, and clinical stage.

## 4 GLIOMA

Guan et al^30^ demonstrated that IKBKE was highly expressed in glioma cell lines and human primary glioma tissues at mRNA and protein levels but that IKBKE expression was not associated with the pathological grade of gliomas by ICH (immunohistochemical) analysis. They testified that the expression of IKBKE was closely associated with the apoptotic markers caspase‐3 and Bcl‐2. IKBKE knockdown in glioma cells decreased Bcl‐2 expression and promoted cleavage and activation of caspase‐3, which suggested that IKBKE induced glioma cell resistance to apoptosis. Subsequently, Li et al^31^ confirmed that the overexpression of IKBKE in gliomas was positively related to the grade of glioma by ICH analysis. Silencing of IKBKE in human glioma cells using siRNA showed significant inhibition of cell growth, migration, and invasion and arrested tumor cells at the G_0_/G_1_ phase. However, notable apoptosis was not observed. Furthermore, when nude mice were treated with IKBKE siRNA in vivo, the tumor growth of established subcutaneous gliomas was significantly attenuated. Lu et al^32^ also demonstrated that IKBKE promoted glioma cell proliferation, migration, invasion, and EMT (epithelial‐mesenchymal transition). A recent study also pointed out that IKBKE influenced glioblastoma chemosensitivity via the NF‐κB pathway.^33^ Collectively, these results suggest that the overexpression of IKBKE plays a critical role in the elevated proliferation and malignant invasion of glioma cells.

## 5 DIGESTIVE SYSTEM TUMOR

Kang et al^34^ compared IKBKE expression in 58 esophageal squamous cell cancers and 58 normal paracancerous tissues by immunohistochemical staining and found that IKBKE was upregulated in 84% of cancer tissues and that an increased degree of IKBKE was not associated with tumor differentiation, depth of invasion or TNM stage. Lee et al^35^ analyzed the expression levels of IKBKE and TBK1 using tissue microarray samples obtained from 1107 gastric cancer patients and found upregulation of IKBKE in 150 samples (13.6%) and upregulation of TBK1 in 38 samples (3.4%). Furthermore, co‐upregulation of IKBKE and TBK1 was identified in 1.5% of cases. In addition, co‐upregulation of IKBKE and TBK1 was associated with differentiated intestinal histology and earlier T stage. In addition, the mean survival time of the IKBKE^+^/TBK1^+^ subgroup was better than that of the other groups, but the difference in prolonged survival time was not statistically significant, mainly due to the small number of patients. Subsequently, Geng et al^36^ testified that IKBKE was overexpressed in gastric cancer by immunohistochemical staining, which was correlated with more advanced disease and poor overall survival of patients. Silencing of IKBKE effectively suppressed the migratory and invasive capabilities of human gastric cancer cells in vitro and tumorigenicity and metastasis in vivo. To analyze IKBKE expression in HCC (hepatocellular carcinoma), Tang et al^37^ found that IKBKE expression was significantly increased in 200 of 222 HCC tissues compared with the adjacent normal tissues. In addition, high expression of IKBKE correlated with short overall survival. Colony formation in HCC cell lines was increased after transfection with IKBKE plasmids, indicating that IKBKE was related to the oncogenesis and progression of HCC. Cheng et al^38^ immunohistochemically evaluated IKBKE expression in 78 PDAC (pancreatic ductal adenocarcinoma) patients and found that IKBKE was overexpressed in 50 (64%) PDAC specimens. Moreover, they also discovered that patients with high expression of IKBKE had a significantly worse prognosis than low‐expression patients. Zubair et al^39^ demonstrated that silencing of IKBKE in PDAC cell lines inhibited the proliferation of tumor cells and the number of colony forming cells and reduced the glucose uptake of tumor cells. In the next experiments in vivo, IKBKE knockdown pancreatic cancer cells, compared with normal pancreatic cancer cells, not only reduced tumor volume but also decreased glucose uptake and suppressed tumor metastasis, suggesting that IKBKE played an important role in the progression of pancreatic cancer. Similarly, Rajurkar et al^40^, ^41^ reported that silencing of IKBKE inhibited PDAC cell proliferation and promoted PDAC apoptosis, thus suppressing the progression of PDAC in vitro. In an in vivo experiment, knockdown of IKBKE also inhibited the initiation and progression of pancreatic tumors in mice. Göktuna et al^42^ found that IKBKE promoted intestinal cell survival and established an inflammatory tumor microenvironment in CRC (colorectal cancer) upon constitutive Wnt activation. The antiapoptotic ability of IKBKE‐depleted cells was decreased after CRC cells were treated with the protease synthesis inhibitor CHX (cycloheximide). Furthermore, IKBKE‐deficient mice had longer survival in response to Wnt‐driven tumors, and this phenomenon was reversed by antibiotics, suggesting that IKBKE controlled the expression of intestinal antimicrobial factors upon constitutive Wnt signaling. Chen et al^43^ showed that IKBKE knockdown could inhibit the proliferative capacity of colorectal cancer cell lines. In a study of drug resistance, Zhao et al^44^ observed that IKBKE was highly expressed in VCR (vincristine)‐resistant colon cancer cells. Thus, IKBKE expression was considered to be associated with the resistance of colon cancer cells to VCR.

## 6 FEMALE REPRODUCTIVE SYSTEM TUMORS

To explore the role of IKBKE in ovarian cancer, Guo et al^45^ demonstrated that IKBKE was significantly overexpressed and activated both in human ovarian cancer cell lines and primary tumors. Of the 96 ovarian cancer specimens examined, 63 had meaningful IKBKE overexpression at both the mRNA and protein levels. Moreover, IKBKE overexpression was associated with late stage, high grade, poor prognosis, and resistance to cisplatin, all of which suggested that IKBKE was a critical mediator of ovarian cancer progression and drug resistance. Hsu et al^46^ surveyed IKBKE expression in 42 primary ovarian carcinomas and 78 metastatic carcinomas. They found that cytoplasmic IKBKE expression was much higher in metastatic carcinomas than in primary carcinomas, and this change was not observed with IKKα and IKKβ. In an in vitro experiment, silencing of IKBKE slightly decreased growth and invasion in ovarian cancer cell lines. Consistently, human xenografts with IKBKE knocked down in mice demonstrated meaningfully decreased growth, aggressiveness, and metastasis. Taken together, IKBKE is a critical coordinator of progression, invasion, and metastasis in ovarian cancer. Colas et al^47^ analyzed 52 carcinoma samples using gene expression screening and found that IKBKE was overexpressed in EC (endometrial carcinoma).

## 7 MALE UROLOGICAL TUMORS

Ghatalia et al^48^ reported that the expression of IKBKE was much higher in metastatic renal clear cell carcinoma tissues than in primary tumors using kinase gene expression profiling analysis. In addition, IKBKE overexpression in metastases was also identified by TCGA (The Cancer Genome Atlas) analysis, which indicated that IKBKE may play a role in the metastasis of renal clear cell carcinoma. Hildebrandt et al^49^ performed a systematic analysis of gene expression for 503 kinases in 93 tumor samples and adjacent normal tissues. They found that the lifespan of patients with high IKBKE expression was shorter than that of patients with low IKBKE expression. Peant et al^50^ reported that the overexpression of IKBKE in hormone‐sensitive prostate cancer cells could induce inflammatory cytokine secretion, such as IL‐6 and IL‐8. Knocking down IKBKE in these cell lines resulted in a significant decrease in IL‐6 secretion. These conclusions suggested that IKBKE supported tumor growth through favouring the chronic inflammation microenvironment, which was necessary for tumor development. Similarly, Seo et al^51^ analyzed the expression of IKBKE in 107 prostate adenocarcinoma tissues by immunohistochemistry and found that IKBKE was overexpressed in 70.1% of prostate cancers, which indicated that IKBKE might play a role in the tumorigenesis of prostate cancers. Subsequently, Peant et al^52^ performed IKBKE immunostaining on 62 hormone‐sensitive (HS) prostate tissues and 31 castration‐resistant (CR) prostate tissues. They observed that cytoplasmic IKBKE staining in HS tumors was higher than that in normal tissues and that cytoplasmic expression of IKBKE in CR tumor tissues was highest in all PC (prostate cancer) tissues. Furthermore, they observed a significant growth delay both in vitro in IKBKE‐silenced prostate cancer cell lines and in vivo in xenografted mice, and they also observed longer survival in IKBKE knockdown xenografted mice.^53^

## 8 LUNG CANCER

Guo et al^54^ examined the expression of IKBKE in 98 NSCLC (non‐small cell lung cancer) samples and demonstrated that 54 tumor samples had elevated IKBKE expression by immunohistochemistry. In addition, smoking was found to be closely related to IKBKE overexpression owing to the two components of cigarettes, nicotine and nicotine‐derived nitrosamine, which were necessary to induce IKBKE expression. In addition, IKBKE was highly expressed in multiple NSCLC cell lines, and knockdown of IKBKE sensitized NSCLC cells to chemotherapy, suggesting that IKBKE might play a pivotal role in the tumorigenesis and drug resistance of NSCLC. Li et al^55^ analyzed IKBKE expression in 288 paraffin‐embedded squamous cell carcinomas of lung (SCCL) specimens. Among them, 204 specimens showed significantly upregulated IKBKE expression compared with that of normal bronchial epithelium. In addition, IKBKE expression in SCCL was significantly associated with smoking status, smoking index, degree of differentiation, and clinical stage. They also analyzed IKBKE mRNA expression by reverse transcription PCR in 66 SCCL specimens and indicated that the mRNA expression level was higher in SCCL than in normal bronchial epithelium. Moreover, the expression of IKBKE mRNA displayed an obvious upward trend with the smoking index. All these data suggested that IKBKE upregulation was positively associated with malignant transformation of human bronchial epithelial cells. Challa et al^56^ found high expression of IKBKE in several NSCLC cell lines and silencing of IKBKE decreased tumor cell proliferation, colony growth and invasion in vitro. Combining IKBKE small molecule inhibitors with MEK inhibitors significantly inhibited the xenograft tumor growth of NSCLC in vivo. Recently, Wang et al^57^ demonstrated that IKBKE+/TBK1+ co‐expression was significant in patients with risk factors and with a recurrent pattern of distant metastasis. Taken together, these findings indicate that IKBKE plays a pivotal role in tumorigenesis and the progression of NSCLC.

## 9 OTHER TUMORS

Zhang et al^58^ demonstrated that silencing of IKBKE significantly decreased proliferation and caused apoptosis of HTLV‐1 (human T‐cell leukemia virus type 1) transformed T lymphocytes. These results indicated a prosurvival role of IKBKE in HTLV‐1‐transformed T cells. Wang et al^59^ reported that IKBKE was implicated in inflammation‐driven tumorigenesis. They found that IKBKE was critically required for tumorigenesis by activating the NF‐κB pathway and that IKBKE expression was drastically upregulated in Kaposi sarcoma‐like lesions and that loss of IKBKE abolished tumor formation. Liu et al showed that IKBKE was overexpressed in AML (acute myeloid leukemia) through database analysis, but it was puzzling that patients with the 10% highest IKBKE expression or AML subtypes with the highest IKBKE expression showed significantly increased overall survival.^60^

## 10 IKBKE IN DIVERSE SIGNALING PATHWAYS ASSOCIATED WITH MALIGNANCY

**Figure 1:**
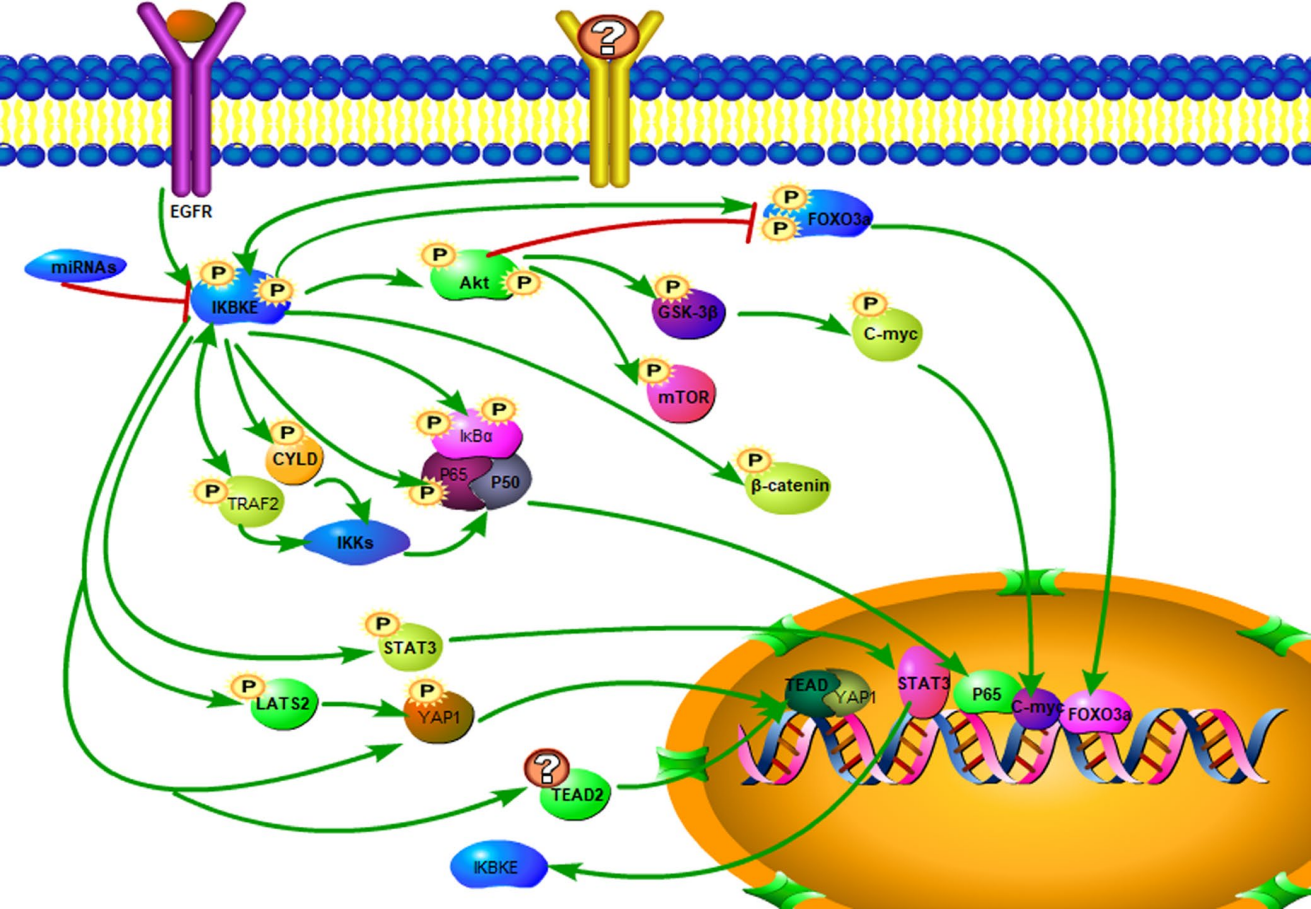
IKBKE played important role in multiple signaling pathways including NF‐κB, Akt, STAT, Hippo, and Wnt/β‐catenin signaling pathways to regulate cancer development

IKBKE plays an important role in multiple signaling pathways, including NF‐κB, Akt, STAT (signal transducer and activator of transcription), Hippo and the Wnt/β‐catenin signaling pathways, and participates in the progression of tumors, inflammation‐driven cancer development, tumor microenvironment regulation and so on (Figure 1). Here, we highlight the association between IKBKE and multiple cytokines, miRNA, and signaling pathways in tumors.

## 11 IKBKE AND NF‐κB PATHWAYS

The cranial NF‐κB signaling pathway is mainly composed of NF‐κB factors and their inhibitors. There are five family member protein monomers: RelA/p65, RelB, c‐Rel, p50/p105, and p52/p100. They form homodimers or heterodimers to affect tumor functions. Under normal circumstances, the NF‐κB factors stay in the cytoplasm combined with IκB family members, such as IκBα, IκBβ, and IκBε, to form a protein complex, which results in NF‐κB retention in the cytoplasm and thus deprivation of transcriptional activation. When the microenvironment of tumor cells is changed to activate the NF‐κB pathway, the IKK (inhibitor of nuclear factor kappa‐B kinase) complex, mainly composed of IKKα, IKKβ, and IKKγ (NEMO), directly phosphorylates IκB family proteins, leading to its polyubiquitination and subsequent degradation in the cytoplasm, thereby releasing NF‐κB homodimers or heterodimers that localize to the nucleus where dimers can target DNA and induce gene translation.^61^, ^62^, ^63^ Early studies have shown that IKBKE could upregulate phosphorylated IκBα at the serine 32 site and serine 36 site to activate the NF‐κB pathway.^1^, ^2^ Harris et al^64^ showed that IKBKE directly phosphorylated the C‐terminal domain of c‐Rel and regulated the nuclear accumulation of c‐Rel to induce the NF‐κB signaling pathway, which was independent of the classical IKK/IκB pathway. In addition, Adli et al^65^ demonstrated that IKBKE was highly expressed in multiple cancer cells, directly phosphorylates p65 at the serine 536 site to enhance NF‐κB transactivation and then promoted the proliferation of cancer cells. Similarly, Buss et al^66^ achieved the same conclusion in their research. Recently, Wang et al^59^ certified that IKBKE promotes NF‐κB subunit RelA (also known as p65) phosphorylation at serine 468, which correlated with NF‐κB activation and inflammatory cytokine expression in Kaposi sarcoma.

In glioma, IKBKE promoted the translocation of NF‐κB transcription factors composed of a heterodimer of p50 and p65 subunits into the nucleus and initiated the transcription of downstream factors of the NF‐κB signaling pathway to accelerate the progression of glioma.^30^, ^31^ Zhang et al^33^ pointed out that PLK4 could directly phosphorylate IKBKE at Ser‐36 to sensitize it, thus activating the NF‐κB pathway to enhance glioblastoma chemotherapy resistance. For breast cancer, IKBKE also promoted the tumorigenesis and progression of tumors by activating the NF‐κB signaling pathway.^24^, ^26^, ^27^, ^67^, ^72^ Similarly, Shen et al^68^ demonstrated that IKBKE directly interacted with and phosphorylated TRAF2 (tumor necrosis factor receptor‐associated factor 2) at serine 11 and then promoted Lys63‐linked TRAF2 ubiquitination to recruit the canonical IKK complex and RIP1, thereby inducing NF‐κB activation and accelerating the malignant transformation of breast cancer cell lines. Recently, Zhou et al^69^ reported that the cIAP1/cIAP2/TRAF2 E3 ubiquitin ligase complex bound to and modified IKBKE by Lys63‐linked polyubiquitination of IKBKE at lysine 30 and lysine 401, which activated IKBKE and promoted its oncogenic functions through the NF‐κB pathway. Furthermore, Hutti et al^70^ identified that IKBKE directly phosphorylated an important tumor suppressor, CYLD (cylindromatosis), at the serine 418 site, thus decreasing the deubiquitinase activity of CYLD and decreasing the suppressive functions of TRAF2 and NEMO, which accelerated NF‐κB activity and promoted breast cancer malignant transformation. A recent study showed that IKBKE could directly phosphorylate RIPK1 in the TNFR1 signaling complex (TNFR1SC) to prevent TNF‐induced cell death independent of NEMO/IKKγ.^71^

## 12 IKBKE‐ AND AKT‐ASSOCIATED PATHWAYS

Xie et al^73^ demonstrated that IKBKE activated AKT by directly phosphorylating AKT at Ser473 and Thr308 and then accelerated the activation of the downstream signaling pathway and promoted the oncogenic function of IKBKE. Meanwhile, this research revealed that the activation of AKT induced by IKBKE required the involvement of PI3K (phosphatidylinositol‐3 kinase). Mahajan et al^74^ reported that the activation of AKT induced by IKBKE did not require the involvement of PI3K. Recently, Zubair et al^39^ demonstrated that IKBKE increased c‐Myc in a manner associated with enhanced signaling through an AKT/GSK3β/c‐Myc phosphorylation cascade that promoted c‐Myc nuclear translocation and then triggered the transcriptional activation of c‐Myc to increase glucose uptake, tumorigenesis and metastasis of PDAC cells. In breast cancer cell lines, Krishnamurthy et al^75^ found that AKT could regulate TNF‐dependent IKBKE expression levels. When AKT2 was knocked down, decreased TNF‐induced IKBKE expression was observed, whereas AKT2 overexpression increased IKBKE expression. Furthermore, they also demonstrated that IKBKE functions downstream of AKT2 to promote breast cancer cell survival. Rajurkar et al^40^ showed that IKBKE promoted the reactivation of AKT postinhibition of mTOR in PDAC (pancreatic ductal adenocarcinoma) cells. Furthermore, Guo et al^76^ reported that IKBKE regulated FOXO3a (forkhead box O3), the downstream AKT pathway, through direct phosphorylation of FOXO3a at serine 644 and then induced its degradation and nuclear‐cytoplasmic translocation. Previous studies had shown that Akt inhibited FOXO3a by phosphorylation of Ser32, Ser253, and Ser315.^77^ However, the activity of FOXO3a‐A3, which had three serine residues converted to alanine residues and could not be phosphorylated by Akt, was inhibited by IKBKE. After phosphorylation by IKBKE, FOXO3a was deprived of the ability to induce tumor cell apoptosis due to its retention and degradation in the cytoplasm, thus accelerating the progression of NSCLC and breast cancer cells.

## 13 IKBKE AND HIPPO PATHWAYS

The Hippo pathway mainly regulates cell proliferation, apoptosis, differentiation, and stemness in response to changes in the intracellular and extracellular microenvironment, including cell contact, cell polarity, mechanotransduction, and G‐protein‐coupled receptor (GPCR) signaling.^78^, ^79^ The core Hippo pathway is composed of a series of serine/threonine kinases, such as MST (mammalian Ste2‐like kinases), LATS (large tumor suppressor kinases), MOB (MOB kinase activator), and SAV (Salvador). Normally, the Hippo pathway is activated. MST directly phosphorylates and activates LATS with the help of SAV. Then, LATS directly phosphorylates and restricts the activity of two transcriptional co‐activators, YAP (Yes‐associated protein) and TAZ (transcriptional coactivator with PDZ binding motif). Phosphorylated YAP1 induced by LATS, mainly at the serine 127 site, is ubiquitinated by 14‐3‐3 and degrades in the cytoplasm.^80^ Recently, Lu et al^32^ discovered that IKBKE promoted the expression of YAP1 and TEAD2 (transcriptional enhancer activation domain 2), which are two important downstream factors of the Hippo pathway in glioma cell lines, thus accelerating the EMT (epithelial‐mesenchymal transition) of cancers. IKBKE could directly combine with YAP1 and TEAD2, which were detected by endogenous IP (immunoprecipitation). Zhang et al^81^ reported that IKBKE increased the expression of YAP1, promoted YAP1 translocation into the nucleus and decreased the expression of p‐YAP1 (Ser127), which was a degradative marker of YAP1, thereby promoting the progression of glioblastoma. Liu et al^82^ demonstrated that direct interaction of IKBKE with LATS induced degradation of LATS, thereby inhibiting the activity of the Hippo pathway and facilitating glioblastoma cell line development.

## 14 IKBKE AND STAT

Recently, Zhang et al^58^ demonstrated that IKBKE played an important role in maintaining the activity of STAT3 in lymphoma cell lines, thereby accelerating lymphoma growth and malignant progression. Interestingly, after silencing IKBKE, the expression levels of IKKα, IKKβ, p65, p50, p100, p52, and p‐p65 were negligibly changed, suggesting that IKBKE had no significant effect on the NF‐κB pathway in lymphoma. Similarly, Barbie et al^27^ confirmed that the overexpression of IKBKE was not only associated with NF‐κB activation by measuring the expression level of p‐p105 at Ser933 but also associated with the activation of STAT3 by analyzing the expression of p‐STAT3 at Thr705. They also confirmed that the activation of NF‐κB and STAT3 induced by IKBKE was associated with the expression of CCL5 (chemokine ligand 5) and promoted tumorigenicity and progression of breast cancer through these pathways. Guo et al^54^ confirmed that IKBKE was a direct downstream target of STAT3 through ChIP (chromatin immunoprecipitation) and luciferase reporter assay analysis. Meanwhile, overexpression of IKBKE induced by STAT increased chemoresistance in NSCLC (non‐small cell lung cancer) cells.

## 15 IKBKE AND EGFR

Williams et al^83^ identified a novel association between IKBKE and EGFR expression (*P* = .0011) using ICH (immunohistochemistry) analysis in breast cancer specimens, and knockdown of IKBKE using siRNA decreases the expression of EGFR. Challa et al^56^ testified that both wild‐type and mutant EGFR directly interacts with IKBKE, whereas only mutant EGFR, which tended to develop resistance to therapeutic EGFR inhibitors, phosphorylated IKBKE on Tyr153 and Tyr179 residues to promote proliferation and invasion of NSCLC in vitro and in vivo.

## 16 IKBKE‐ AND β‐CATENIN‐ASSOCIATED PATHWAYS

Chen et al^43^ testified that IKBKE physically interacted with β‐catenin by co‐IP (co‐immunoprecipitation) and that IKBKE phosphorylated β‐catenin, probably at Ser680 and Ser681, to restrain its hyperactivation, thereby promoting colorectal cancer (CRC) cell proliferation. In addition, Göktuna et al^42^ discovered that IKBKE established a proinflammatory signature in the intestine upon constitutive Wnt signaling and that genetic ablation of IKBKE in β‐catenin‐driven models of intestinal cancer‐reduced tumor incidence and consequently extended survival. These results indicated that IKBKE influenced the β‐catenin‐associated pathway; however, the specific mechanism remained to be elucidated.

## 17 IKBKE AND MICRORNAs

Since IKBKE has become an increasingly important therapeutic target for numerous malignancies, a series of microRNAs have been identified as vital regulators to control the expression of IKBKE, thus promoting tumor progression. Yuan et al^84^ testified through luciferase reporter assay analysis that miR‐let‐7b/i suppressed glioblastoma cell invasion and migration by targeting IKBKE to reduce IKBKE expression. Similarly, Zhang et al^81^ discovered that miR‐let‐7b/i could decrease the expression of IKBKE, while IKBKE also decreased the expression of miR‐let‐7b/i through inducing YAP1, thus forming a miR‐let‐7/IKBKE/YAP1 regulatory loop to regulate glioblastoma cell growth. Tang et al^37^ demonstrated that miR‐4458 targeted IKBKE to reduce its expression in human hepatocellular carcinoma to suppress cancer cell growth. Furthermore, Tang et al^29^ identified IKBKE as a direct target of miR‐16, and overexpression of miR‐16 promoted taxol‐induced cytotoxicity and apoptosis in breast cancer cells via decreasing IKBKE expression. Wu et al^67^ demonstrated that miR‐200b downregulates IKBKE expression via directly binding to its 3ʹ‐UTR, thus suppressing NF‐κB activation to inhibit cancer progression. A recent study also pointed out that miR‐155 could lower the expression of IKBKE.^70^

## 18 IKBKE AND OTHER PATHWAYS

Li et al^28^ demonstrated that IKBKE interacted with ERα‐36, which lacks intrinsic transcriptional activity and, in contrast with ERα, mediated mainly nongenomic estrogen signaling; it also has increased expression in breast cancer cells. Then, ERα‐36 promoted cell proliferation through the MAPK/ERK pathway in ER‐negative breast cancers. Peant et al^53^ also confirmed that IKBKE overexpression induced NF‐κB‐independent stimulation of IL‐6 expression through the activation and nuclear translocation of transcription factor C/EBP‐β in prostate cancer cells. Meanwhile, Zhu et al^85^ showed that the upregulation of IKBKE promoted KRAS‐driven tumorigenesis and metastasis by regulating CCL5 and IL‐6 in NSCLC cell lines. Liu et al reported that IKBKE could phosphorylate YB‐1, an oncogenic gene regulator, and then increased its activity to enhance MYC gene transcription.^60^ Rajurkar et al^40^ demonstrated that the Gli transcription factor upregulated IKBKE expression at both the mRNA and protein levels, thereby increasing the activity of the NF‐κB pathway to maintain the transformation and survival of Kras‐induced pancreatic cancer cells. More recently, Rajurkar et al^41^ confirmed that IKBKE was co‐expressed with Gli and that IKBKE promoted the nuclear translocation of Gli as measured by immunofluorescence staining and western blotting. Li et al demonstrated that MEF2D, a member of the MEF2 family, could directly target the IKBKE promoter to control its translation to enhance tumor chemotherapeutic resistance in ovarian carcinoma.^86^ Cheng et al^87^ demonstrated that zinc finger protein (ZNF382), which functioned as a tumor suppressor, was methylated in multiple primary tumors, including nasopharyngeal, esophageal, colon, gastric, and breast cancers, thereby suppressing the NF‐κB pathway and AP‐1 signaling through downregulating IKBKE.

## 19 IKBKE: A NEW STRATEGIC THERAPEUTIC TARGET

As the regulatory mechanism of IKBKE in tumors has gradually been revealed in recent years, its critical role in tumorigenesis and development of malignant tumors has been increasingly recognized. The research of small molecule inhibitors targeting IKBKE has also become a hot topic.

Using an in vitro kinase assay, Zhu et al^85^ indicated that CYT387, a small molecule inhibitor, not only inhibits JAK/STAT signaling pathway activation but also inhibits IKBKE kinase activity. CYT387 significantly reduced tumorigenesis of NSCLC through interrupting the IKBKE‐induced autocrine cytokine feedback loop required for KRAS‐driven lung tumorigenesis. Furthermore, Barbie et al^27^ found that treatment of IKBKE‐driven breast cancer cells with CYT387, a potent inhibitor of TBK1/IKBKE and JAK signaling, reduced the proliferation and migration of TNBC cells, whereas inhibition of JAK alone does not. A combination of CTY387 with a MEK inhibitor is definitely an effective therapy for abrogating the growth of patient‐derived xenografts. In addition, Hu et al^88^ demonstrated that CYT387 was highly effective in combination with EGFR inhibitor against NSCLC tumors, particularly with EGFR inhibitor tumors that have intrinsic resistance. Liu also showed that CYT387 reduced the viability and clonogenicity of primary AML cells and demonstrated efficacy in a murine model of AML.^60^

Recently, Li et al^89^ reviewed that targeting IKBKE with three TBK1/IKBKE dual inhibitors, including WO2009032861, SAR and Domainex, could powerfully inhibit cell viability and tumor development in human breast, prostate, and oral cancers both in vivo and in vitro. These inhibitors’ anticancer functions were partially owing to their suppression of TBK1/IKBKE‐mediated AKT phosphorylation and VEGF (vascular endothelial cell growth factor) expression. Liu et al^90^ showed that MCCK1, a specific and effective IKKε inhibitor, enhanced the anticancer effect of temozolomide in glioblastoma, suggesting that IKKε was associated with chemotherapeutic resistance of glioblastoma.

In addition to these studies, emerging evidence has demonstrated that amlexanox, a small molecule regulator used to treat ulcers and asthma, can selectively inhibit IKBKE kinase activation through competing with IKBKE on the ATP‐binding site.^91^ Furthermore, Challa et al^56^ confirmed that combining amlexanox with the MEK inhibitor AZD6244 inhibits the in vivo growth of xenografted NSCLC cells targeting activating EGFR mutations, including EGFR^T790M^. IKBKE may be a direct target for reversing EGFR‐TKI‐resistance in NSCLC. In addition, Liu et al^82^ certified that amlexanox, as an IKBKE inhibitor, suppresses glioblastoma cell growth and development in vivo and in vitro. More recently, Cheng et al^92^ showed that amlexanox inhibits the mobility, migration, metastasis, and EMT of prostate tumors in vitro and in vivo by the IKBKE/TBK1/NF‐κB pathway.

## 20 CONCLUSIONS AND PERSPECTIVES

Recently, IKBKE was defined as a new oncogene in breast cancer and was subsequently found to be overexpressed in various kinds of tumors, including female reproductive system tumors, lung cancer (especially NSCLC), gastrointestinal tumors, male urological tumors and gliomas. Expanded research revealed that the tumorigenic functions of IKBKE were not limited to the NF‐κB signaling pathway but also extended to other signaling pathways, including AKT, STAT3, Hippo, and EGFR, mainly binding a large crosstalk network with numerous cytokines. Meanwhile, an increasing number of studies have suggested that the tumorigenic effect of IKBKE is related to its role in facilitating the secretion of associated inflammatory cytokines, thereby affecting the tumor microenvironment and accelerating tumor development.

Overall, IKBKE is closely related to tumorigenesis and the development of cancers. In addition, emerging evidence demonstrated that combining a small molecule inhibitor of IKBKE with other inhibitors suppresses the growth of xenografted tumor cells. In the future treatment of malignant tumors, IKBKE may be a therapeutic target and an important candidate for clinical evaluation.

## CONFLICT OF INTEREST

The authors disclose no conflict of interest.

## References

[1] Shimada T , Kawai T , Takeda K , et al. IKK‐i, a novel lipopolysaccharide inducible kinase that is related to IkappaB kinases. Int Immunol. 1999;11(8):1357‐1362. 10421793 10.1093/intimm/11.8.1357

[2] Peters RT , Liao SM , Maniatis T . IKKepsilon is part of a novel PMA‐inducible IkappaB kinase complex. Mol Cell. 2000;5(3):513‐522. 10882136 10.1016/s1097-2765(00)80445-1

[3] Chien Y , Kim S , Bumeister R , et al. RalB GTPase‐mediated activation of the IkappaB family kinase TBK1 couples innate immune signaling to tumor cell survival. Cell. 2006;127(1):157‐170. 17018283 10.1016/j.cell.2006.08.034

[4] Ikeda F , Hecker CM , Rozenknop A , et al. Involvement of the ubiquitin‐likedomain of TBK1/IKK‐i kinases in regulation of IFN‐inducible genes. EMBO J. 2007;26(14):3451‐3462. 17599067 10.1038/sj.emboj.7601773 PMC1933404

[5] Kawai T , Akira S . Signaling to NF‐kappaB by Toll‐like receptors. Trends MolMed. 2007;13(11):460‐469. 10.1016/j.molmed.2007.09.002 18029230

[6] Chau TL , Gioia R , Gatot JS , et al. Are the IKKs and IKK‐related kinases TBK1and IKK‐epsilon similarly activated? Trends Biochem Sci. 2008;33(4):171‐180. 18353649 10.1016/j.tibs.2008.01.002

[7] Verhelst K , Verstrepen L , Carpentier I , Beyaert R . IκB kinase ε (IKKε): a therapeutic target in inflammation and cancer. Biochem Pharmacol. 2013;85(7):873‐880. 23333767 10.1016/j.bcp.2013.01.007 PMC7111187

[8] Pomerantz JL , Baltimore D . NF‐kappaB activation by a signaling complex containing TRAF2, TANK and TBK1, a novel IKK‐related kinase. EMBO J. 1999;18(23):6694‐6704. 10581243 10.1093/emboj/18.23.6694 PMC1171732

[9] Nomura F , Kawai T , Nakanishi K , Akira S . NF‐kappaB activation through IKK‐i‐dependent I‐TRAF/TANK phosphorylation. Genes Cells. 2000;5(3):191‐202. 10759890 10.1046/j.1365-2443.2000.00315.x

[10] Gatot JS , Gioia R , Chau TL , et al. Lipopolysaccharide‐mediated interferon regulatory factor activation involves TBK1‐IKK epsilon‐dependent Lys(63)‐linked polyubiquitination and phosphorylation of TANK/I‐TRAF. J Biol Chem. 2007;282(43):31131‐31146. 17823124 10.1074/jbc.M701690200

[11] Fujita F , Taniguchi Y , Kato T , et al. Identification of NAP1, a regulatory subunit of IkappaB kinase‐related kinases that potentiates NF‐kappaB signaling. Mol Cell Biol. 2003;23(21):7780‐7793. 14560022 10.1128/MCB.23.21.7780-7793.2003 PMC207563

[12] Ryzhakov G , Randow F . SINTBAD, a novel component of innate antiviral immunity, shares a TBK1‐binding domain with NAP1 and TANK. EMBO J. 2007;26(13):3180‐3190. 17568778 10.1038/sj.emboj.7601743 PMC1914091

[13] Koop A , Lepenies I , Braum O , et al. Novel splice variants of human IKKε negatively regulate IKKε‐induced IRF3 and NF‐κB activation. Eur J Immunol. 2011;41(1):224‐234. 21182093 10.1002/eji.201040814

[14] Nakatsu Y , Matsuoka M , Chang T‐H , et al. Functionally distinct effects of the C‐terminal regions of IKKε and TBK1 on type I IFN production. PLoS ONE. 2014;9(4):e94999. 24722368 10.1371/journal.pone.0094999 PMC3983252

[15] Clément JF , Meloche S , Servant MJ . The IKK‐related kinases: from innate immunity to oncogenesis. Cell Res. 2008;18(9):889‐899. 19160540 10.1038/cr.2008.273

[16] Tenoever BR , Ng SL , Chua MA , et al. Multiple functions of the IKK‐related kinase IKKepsilon in interferon‐mediated antiviral immunity. Science. 2007;315(5816):1274‐1278. 17332413 10.1126/science.1136567

[17] Hemmi H , Takeuchi O , Sato S , et al. The roles of two IkappaB kinase‐related kinases in lipopolysaccharide and double stranded RNA signaling and viral infection. J Exp Med. 2004;199(12):1641‐1650. 15210742 10.1084/jem.20040520 PMC2212809

[18] Fujii K , Nakamura S , Takahashi K , Inagaki F . Systematic characterization by mass spectrometric analysis of phosphorylation sites in IRF‐3 regulatory domain activated by IKK‐i. J Proteomics. 2010;73(6):1196‐1203. 20170763 10.1016/j.jprot.2010.02.009

[19] Sato M , Suemori H , Hata N , et al. Distinct and essential roles of transcription factors IRF‐3 and IRF‐7 in response to viruses for IFN‐alpha/beta gene induction. Immunity. 2000;13(4):539‐548. 11070172 10.1016/s1074-7613(00)00053-4

[20] Diani E , Avesani F , Bergamo E , Cremonese G , Bertazzoni U , Romanelli MG . HTLV‐1 Tax protein recruitment intoIKKε and TBK1 kinase complexes enhances IFN‐I expression. Virology. 2015;476:92‐99. 25531185 10.1016/j.virol.2014.12.005

[21] McWhirter SM , Fitzgerald KA , Rosains J , Rowe DC , Golenbock DT , Maniatis T . IFN‐regulatory factor 3‐dependent gene expression is defective in Tbk1‐deficient mouse embryonic fibroblasts. Proc Natl Acad Sci USA. 2004;101(1):233‐238. 14679297 10.1073/pnas.2237236100 PMC314168

[22] Perry AK , Chow EK , Goodnough JB , Yeh WC , Cheng G . Differential requirement for TANK‐binding kinase‐1 in type I interferon responses totoll‐like receptor activation and viral infection. J Exp Med. 2004;199(12):1651‐1658. 15210743 10.1084/jem.20040528 PMC2212814

[23] Siednienko J , Gajanayake T , Fitzgerald KA , Moynagh P , Miggin SM . Absence of MyD88 results in enhanced TLR3‐dependent phosphorylation of IRF3 and increased IFN‐β and RANTES production. J Immunol. 2011;186(4):2514‐2522. 21248248 10.4049/jimmunol.1003093

[24] Eddy SF , Guo S , Demicco EG , et al. Inducible IkappaB kinase/IkappaB kinase epsilon expression is induced by CK2 and promotes aberrant nuclear factor‐kappaB activation in breast cancer cells. Cancer Res. 2005;65(24):11375‐11383. 16357145 10.1158/0008-5472.CAN-05-1602

[25] Boehm JS , Zhao JJ , Yao J , et al. Integrative genomic approaches identify IKBKE as a breast cancer oncogene. Cell. 2007;129(6):1065‐1079. 17574021 10.1016/j.cell.2007.03.052

[26] Qin B , Cheng K . Silencing of the IKKε gene by siRNA inhibits invasiveness and growth of breast cancer cells. Breast Cancer Res. 2010;12(5):R74. 20863366 10.1186/bcr2644 PMC3096963

[27] Barbie TU , Alexe G , Aref AR , et al. Targeting an IKBKE cytokine network impairs triple‐negative breast cancer growth. J Clin Invest. 2014;124(12):5411‐5423. 25365225 10.1172/JCI75661 PMC4348940

[28] Li Q , Sun H , Zou J , et al. Increased expression of estrogen receptor α‐36 by breast cancer oncogene IKKε promotes growth of ER‐negative breast cancer cells. Cell Physiol Biochem. 2013;31(6):833‐841. 23816933 10.1159/000350101

[29] Tang X , Jin L , Cao P , et al. MicroRNA‐16 sensitizes breast cancer cells to paclitaxel through suppression of IKBKB expression. Oncotarget. 2016;7(17):23668‐23683. 26993770 10.18632/oncotarget.8056 PMC5029655

[30] Guan H , Zhang H , Cai J , et al. IKBKE is over‐expressed in glioma and contributes to resistance of glioma cells to apoptosis via activating NF‐κB. J Pathol. 2011;223(3):436‐445. 21171089 10.1002/path.2815

[31] Li H , Chen L , Zhang A , et al. Silencing of IKKε using siRNA inhibits proliferation and invasion of glioma cells in vitro and in vivo. Int J Oncol. 2012;41(1):169‐178. 22552702 10.3892/ijo.2012.1452

[32] Lu J , Yang Y , Guo G , et al. IKBKE regulates cell proliferation and epithelial‐mesenchymal transition of human malignant glioma via the Hippo pathway. Oncotarget. 2017;8(30):49502‐49514. 28548934 10.18632/oncotarget.17738 PMC5564784

[33] Zhang Z , Wang Z , Huang K , et al. PLK4 is a determinant of temozolomide sensitivity through phosphorylation of IKBKE in glioblastoma. Cancer Lett. 2019;443:91‐107. 30529153 10.1016/j.canlet.2018.11.034

[34] Kang MR , Kim MS , Kim SS , et al. NF‐kappaB signalling proteins p50/p105, p52/p100, RelA, and IKKepsilon are over‐expressed in oesophageal squamous cell carcinomas. Pathology. 2009;41(7):622‐625. 20001340 10.3109/00313020903257756

[35] Lee SE , Hong M , Cho J , Lee J , Kim KM . IKKε and TBK1 expression in gastric cancer. Oncotarget. 2017;8(10):16233‐16242. 27145266 10.18632/oncotarget.9069 PMC5369959

[36] Geng B , Zhang C , Wang C , et al. IκB‐kinase‐ε in the tumor microenvironment is essential for the progression of gastric cancer. Oncotarget. 2017;8(43):75298‐75307. 29088866 10.18632/oncotarget.20778 PMC5650421

[37] Tang D , Sun B , Yu H , Yang Z , Zhu L . Tumor‐suppressing effect of miR‐4458 on human hepatocellular carcinoma. Cell Physiol Biochem. 2015;35(5):1797‐1807. 25833000 10.1159/000373991

[38] Cheng A , Guo J , Henderson‐Jackson E , et al. IκB Kinase ε expression in pancreatic ductal adenocarcinoma. Am J Clin Pathol. 2011;136(1):60‐66. 21685032 10.1309/AJCP2JJGYNIUAS2V PMC4644942

[39] Zubair H , Azim S , Srivastava SK , et al. Glucose metabolism reprogrammed by overexpression of IKKε promotes pancreatic tumor growth. Cancer Res. 2016;76(24):7254‐7264. 27923829 10.1158/0008-5472.CAN-16-1666 PMC5161695

[40] Rajurkar M , Dang K , Fernandez‐Barrena MG , et al. IKBKE is required during KRAS‐induced pancreatic tumorigenesis. Cancer Res. 2017;77(2):320‐329. 28069799 10.1158/0008-5472.CAN-15-1684 PMC5243176

[41] Rajurkar M , De Jesus‐Monge WE , Driscoll DR , et al. The activity of Gli transcription factors is essential for Kras‐induced pancreatic tumorigenesis. Proc Natl Acad Sci USA. 2012;109(17):E1038‐E1047. 22493246 10.1073/pnas.1114168109 PMC3340052

[42] Göktuna SI , Shostak K , Chau TL , et al. The prosurvival IKK‐related kinase IKKε integrates LPS and IL17A signaling cascades to promote Wnt‐dependent tumor development in the intestine. Cancer Res. 2016;76(9):2587‐2599. 26980769 10.1158/0008-5472.CAN-15-1473

[43] Chen J , Zhao J , Chen X , et al. Hyper activation of β‐catenin signalling induced by IKKε inhibition thwarts colorectal cancer cell proliferation. Cell Prolif. 2017;50(4). 10.1111/cpr.12350 PMC6529108 28523736

[44] Zhao C‐P , Xu Z‐J , Guo Q , Li Y‐X , Gao X‐Z , Peng Y‐Y . Overexpression of suppressor of IKBKE 1 isassociated with vincristine resistance in colon cancer cells. Biomed Rep. 2016;5(5):585‐588. 27882221 10.3892/br.2016.759 PMC5103677

[45] Guo J‐P , Shu S‐K , He L , et al. Deregulation of IKBKE is associated with tumor progression, poor prognosis, and cisplatin resistance in ovarian cancer. Am J Pathol. 2009;175(1):324‐333. 19497997 10.2353/ajpath.2009.080767 PMC2708818

[46] Hsu S , Kim M , Hernandez L , et al. IKK‐ε coordinates invasion and metastasis of ovarian cancer. Cancer Res. 2012;72(21):5494‐5504. 22942254 10.1158/0008-5472.CAN-11-3993 PMC3488159

[47] Colas E , Perez C , Cabrera S , et al. Molecular markers of endometrial carcinoma detected in uterine aspirates. Int J Cancer. 2011;129(10):2435‐2444. 21207424 10.1002/ijc.25901

[48] Ghatalia P , Yang ES , Lasseigne BN , et al. Kinase gene expression profiling of metastatic clear cell renal cell carcinoma tissue identifies potential new THERAPEUTIC TARGETS. PLoS ONE. 2016;11(8):e0160924. 27574806 10.1371/journal.pone.0160924 PMC5004806

[49] Hildebrandt MAT , Tan W , Tamboli P , et al. Kinome expression profiling identifies IKBKE as a predictor of overall survival in clear cell renal cell carcinoma patients. Carcinogenesis. 2012;33(4):799‐803. 22266464 10.1093/carcin/bgs018 PMC3324439

[50] Péant B , Diallo JS , Dufour F , et al. Over‐expression of IkappaB‐kinase‐epsilon (IKKepsilon/IKKi) induces secretion of inflammatory cytokines in prostate cancer cell lines. Prostate. 2009;69(7):706‐718. 19170126 10.1002/pros.20912

[51] Seo SI , Song SY , Kang MR , et al. Immunohistochemical analysis of NF‐kappaB signaling proteins IKKepsilon, p50/p105, p52/p100 and RelA inprostate cancers. APMIS. 2009;117(8):623‐628. 19664134 10.1111/j.1600-0463.2009.02506.x

[52] Péant B , Forest V , Trudeau V , Latour M , Mes‐Masson A‐M , Saad F . IκB‐Kinase‐ε (IKKε/IKKi/IκBKε) expression and localization in prostate cancer tissues. Prostate. 2011;71(10):1131‐1138. 21271611 10.1002/pros.21329

[53] Péant B , Gilbert S , Le Page C , et al. IκB‐Kinase‐epsilon (IKKε) over‐expression promotes the growth of prostate cancer through the C/EBP‐β dependent activation of IL‐6 gene expression. Oncotarget. 2017;8(9):14487‐14501. 27577074 10.18632/oncotarget.11629 PMC5362420

[54] Guo J , Kim D , Gao J , et al. IKBKE is induced by STAT3 and tobacco carcinogen and determines chemosensitivity in non‐small cell lung cancer. Oncogene. 2013;32(2):151‐159. 22330135 10.1038/onc.2012.39 PMC4109158

[55] Li W , Chen Y , Zhang J , et al. IKBKE upregulation is positively associated with squamous cell carcinoma of the lung in vivo and malignant transformation of human bronchial epithelial cells in vitro. Med Sci Monit. 2015;21:1577‐1586. 26025939 10.12659/MSM.893815 PMC4461048

[56] Challa S , Guo J‐P , Ding X , et al. IKBKE is a substrate of EGFR and a therapeutic target in non‐small cell lung cancer with activating mutations of EGFR. Cancer Res. 2016;76(15):4418‐4429. 27287717 10.1158/0008-5472.CAN-16-0069 PMC4970891

[57] Wang X , Teng F , Lu J , et al. Expression and prognostic role of IKBKE and TBK1 in stage I non‐small cell lung cancer. Cancer Manag Res. 2019;11:6593‐6602. 31406474 10.2147/CMAR.S204924 PMC6642623

[58] Zhang H , Chen L , Cai SH , Cheng H . Identification of TBK1 and IKKε, the non‐canonical IκB kinases, as crucial pro‐survival factors in HTLV‐1‐transformed T lymphocytes. Leuk Res. 2016;46:37‐44. 27123832 10.1016/j.leukres.2016.04.012 PMC4899189

[59] Wang YI , Xiaolu LU , Zhu L , et al. IKK epsilon kinase is crucial for viral G protein‐coupled receptor tumorigenesis. Proc Natl Acad Sci USA. 2013;110(30):12498. 10.1073/pnas.1219829110 PMC3703966 23771900

[60] Liu S , Marneth AE , Alexe G , et al. The kinases IKBKE and TBK1 regulate MYC‐dependent survival pathways through YB‐1 in AML and are targets for therapy. Blood Adv. 2018;2(23):3428‐3442. 30504235 10.1182/bloodadvances.2018016733 PMC6290107

[61] Baldwin AS . Regulation of cell death and autophagy by IKK and NF‐κB:critical mechanisms in immune function and cancer. Immunol Rev. 2012;246(1):327‐345. 22435564 10.1111/j.1600-065X.2012.01095.x

[62] Sethi G , Sung B , Aggarwal BB . Nuclear factor‐kappaB activation: from benchto bedside. Exp Biol Med (Maywood). 2008;233(1):21‐31. 18156302 10.3181/0707-MR-196

[63] Mitchell S , Vargas J , Hoffmann A . Signaling via the NF‐κB system. WileyInterdiscip Rev Syst Biol Med. 2016;8(3):227‐241. 10.1002/wsbm.1331 PMC8363188 26990581

[64] Harris J , Olière S , Sharma S , et al. Nuclear accumulation of cRel following C‐terminal phosphorylation by TBK1/IKK epsilon. J Immunol. 2006;177(4):2527‐2535. 16888014 10.4049/jimmunol.177.4.2527

[65] Adli M , Baldwin AS . IKK‐i/IKKepsilon controls constitutive, cancer cell‐associated NF‐kappaB activity via regulation of Ser‐536 p65/RelA phosphorylation. J Biol Chem. 2006;281(37):26976‐26984. 16840782 10.1074/jbc.M603133200

[66] Buss H , Dörrie A , Schmitz ML , Hoffmann E , Resch K , Kracht M . Constitutive and interleukin‐1‐inducible phosphorylation of p65 NF‐{kappa}B at serine 536 is mediated by multiple protein kinases including I{kappa}B kinase (IKK)‐{alpha}, IKK{beta}, IKK{epsilon}, TRAF family member‐associated (TANK)‐binding kinase 1 (TBK1), and an unknown kinase and couples p65 to TATA‐binding protein‐associated factor II31‐mediated interleukin‐8 transcription. J Biol Chem. 2004;279(53):55633‐55643. 15489227 10.1074/jbc.M409825200

[67] Wu H , Wang G , Wang Z , An S , Ye P , Luo S . A negative feedback loop between miR‐200b and the nuclear factor‐κB pathway via IKBKB/IKK‐β in breast cancer cells. FEBS J. 2016;283(12):2259‐2271. 26433127 10.1111/febs.13543

[68] Shen RR , Zhou AY , Kim E , et al. IκB kinase ε phosphorylates TRAF2 to promote mammary epithelial cell transformation. Mol Cell Biol. 2012;32(23):4756‐4768. 23007157 10.1128/MCB.00468-12 PMC3497603

[69] Zhou AY , Shen RR , Kim E , et al. IKKε‐mediated tumorigenesis requires K63‐linked polyubiquitination by a cIAP1/cIAP2/TRAF2 E3 ubiquitin ligase complex. Cell Rep. 2013;3(3):724‐733. 23453969 10.1016/j.celrep.2013.01.031 PMC4135466

[70] Hutti JE , Shen RR , Abbott DW , et al. Phosphorylation of the tumor suppressor CYLD by the breast cancer oncogene IKKepsilon promotes cell transformation. Mol Cell. 2009;34(4):461‐472. 19481526 10.1016/j.molcel.2009.04.031 PMC2746958

[71] Lafont E , Draber P , Rieser E , et al. TBK1 and IKKε prevent TNF‐induced cell death by RIPK1 phosphorylation. Nat Cell Biol. 2018;20(12):1389‐1399. 30420664 10.1038/s41556-018-0229-6 PMC6268100

[72] Harquail J , LeBlanc N , Landry C , Crapoulet N , Robichaud GA . Pax‐5 inhibits NF‐κB activity in breast cancer cells through IKKε and miRNA‐155 effectors. J Mammary Gland Biol Neoplasia. 2018;23(3):177‐187. 30032344 10.1007/s10911-018-9404-4

[73] Xie X , Zhang D , Zhao B , et al. IkappaB kinase epsilon and TANK‐binding kinase 1 activate AKT by direct phosphorylation. Proc Natl Acad Sci USA. 2011;108(16):6474‐6479. 21464307 10.1073/pnas.1016132108 PMC3081021

[74] Mahajan K , Mahajan NP . PI3K‐independent AKT activation in cancers: a treasure trove for novel therapeutics. J Cell Physiol. 2012;227(9):3178‐3184. 22307544 10.1002/jcp.24065 PMC3358464

[75] Krishnamurthy S , Basu A . Regulation of IKKε expression by Akt2 isoform. Genes Cancer. 2011;2(11):1044‐1050. 22737270 10.1177/1947601912444604 PMC3379568

[76] Guo J‐P , Tian W , Shu S , Xin YU , Shou C , Cheng JQ . IKBKE phosphorylation and inhibition of FOXO3a: a mechanism of IKBKE oncogenic function. PLoS ONE. 2013;8(5):e63636. 23691078 10.1371/journal.pone.0063636 PMC3653944

[77] Brunet A , Bonni A , Zigmond MJ , et al. Akt promotes cell survival by phosphorylating and inhibiting a Forkhead transcription factor. Cell. 1999;96(6):857–868. 10102273 10.1016/s0092-8674(00)80595-4

[78] Yu FX , Guan KL . The Hippo pathway: regulators and regulations. Genes Dev. 2013;27:355‐371. 23431053 10.1101/gad.210773.112 PMC3589553

[79] Yu FX , Zhao B , Guan KL . Hippo pathway in organ size control, tissue homeostasis, and cancer. Cell. 2015;163:811‐828. 26544935 10.1016/j.cell.2015.10.044 PMC4638384

[80] Moroishi T , Hansen CG , Guan KL . The emerging roles of YAP and TAZ in cancer. Nat Rev Cancer. 2015;15:73‐79. 25592648 10.1038/nrc3876 PMC4562315

[81] Zhang Z , Lu J , Guo G , et al. IKBKE promotes glioblastoma progression by establishing the regulatory feedback loop of IKBKE/YAP1/miR‐Let‐7b/i. Tumour Biol. 2017;39:1010428317705575. 28677425 10.1177/1010428317705575

[82] Liu Y , Lu J , Zhang Z , et al. Amlexanox, a selective inhibitor of IKBKE, generates anti‐tumoral effects by disrupting the Hippo pathway in human glioblastoma cell lines. Cell Death Dis. 2017;8(8):e3022. 29048430 10.1038/cddis.2017.396 PMC5596579

[83] Williams V , Grosset A‐A , Zamorano Cuervo N , et al. Detection of IKKε by immunohistochemistry in primary breast cancer: association with EGFR expression and absence of lymph node metastasis. BMC Cancer. 2017;17(1):356. 28532474 10.1186/s12885-017-3321-6 PMC5441089

[84] Tian Y , Hao S , Ye M , et al. MicroRNAs let‐7b/i suppress human glioma cell invasion and migration by targeting IKBKE directly. Biochem Biophys Res Commun. 2015;458(2):307‐312. 25656572 10.1016/j.bbrc.2015.01.105

[85] Zhu Z , Aref AR , Cohoon TJ , et al. Inhibition of KRAS‐driven tumorigenicity by interruption of an autocrine cytokine circuit. Cancer Discov. 2014;4:452‐465. 24444711 10.1158/2159-8290.CD-13-0646 PMC3980023

[86] Li X , Zhang Y , Chai X , et al. Overexpression of MEF2D contributes to oncogenic malignancy and chemotherapeutic resistance in ovarian carcinoma. Am J Cancer Res. 2019;9(5):887‐905. 31218100 PMC6556600

[87] Cheng Y , Geng H , Cheng SH , et al. KRAB zinc finger protein ZNF382 is a pro‐apoptotic tumor suppressor that represses multiple oncogenes and is commonly silenced in multiple carcinomas. Cancer Res. 2010;70(16):6516‐6526. 20682794 10.1158/0008-5472.CAN-09-4566

[88] Hu Y , Dong XZ , Liu X , Liu P , Chen YB . Enhanced antitumour activity of cetuximab in combination with the jak inhibitor CYT387 against non‐small‐cell lung cancer with various genotypes. Mol Pharm. 2016;13(2):689‐697. 26685983 10.1021/acs.molpharmaceut.5b00927

[89] Li J , Huang J , Jeong JH , et al. Selective TBK1/IKKi dual inhibitors swith anticancer potency. Int J Cancer. 2014;134(8):1972‐1980. 24150799 10.1002/ijc.28507 PMC3947486

[90] Liu T , Li A , Xu Y , Xin Y . MCCK1 enhances the anticancer effect of temozolomide in attenuating the invasion, migration and epithelial‐mesenchymal transition of glioblastoma cells in vitro and in vivo. Cancer Med. 2019;8(2):751‐760. 30656846 10.1002/cam4.1951 PMC6382719

[91] Reilly SM , Chiang S‐H , Decker SJ , et al. An inhibitor of the protein kinases TBK1 and IKK‐ɛ improves obesity‐related metabolic dysfunctions in mice. Nat Med. 2013;19(3):313‐321. 23396211 10.1038/nm.3082 PMC3594079

[92] Cheng C , Ji Z , Sheng Y , et al. Aphthous ulcer drug inhibits prostate tumor metastasis by targeting IKKɛ/TBK1/NF‐κB signaling. Theranostics. 2018;8(17):4633‐4648. 30279728 10.7150/thno.26687 PMC6160770

